# Current trends of minimally invasive therapy for cholecystocholedocholithiasis

**DOI:** 10.3389/fmed.2023.1277410

**Published:** 2023-12-13

**Authors:** Anna Cominardi, Giovanni Aragona, Gaetano Cattaneo, Gian Arzù, Patrizio Capelli, Filippo Banchini

**Affiliations:** ^1^Gastroenterology and Digestive Endoscopy Unit, Hospital of Piacenza, Piacenza, Italy; ^2^Emergency Surgery Unit, Hospital of Piacenza, Piacenza, Italy; ^3^General Surgery Unit, Hospital of Piacenza, Piacenza, Italy

**Keywords:** cholecystocholedocholithiasis, endoscopic ultrasound-guided drainage, cholangioscopy, endoscopic retrograde cholangiopancreatography, gallbladder drainage

## Abstract

**Introduction:**

The minimally invasive approach of endoscopic ultrasound (EUS)-guided procedures for cholecystocholedocholithiasis, such as EUS-guided gallbladder drainage (EUS-GBD), EUS-guided rendezvous (EUS-RV), and EUS-guided biliary drainage (EUS-BD), is affirmed as an effective treatment for patients with acute cholecystitis (AC) who are unfit for surgery and for patients with common bile duct stones (CBDSs) who have experienced a previous ERCP failure. Furthermore, in cases of difficult CBDS extraction during endoscopic retrograde cholangiopancreatography (ERCP), cholangioscopy-guided electrohydraulic lithotripsy (CS-EHL) has showed optimal results. The main objective of our study was to evaluate the effectiveness of EUS-GBD and percutaneous gallbladder drainage (PT-GBD) in patients with AC who are unfit for surgery. We also aimed to evaluate the efficacy of EUS-GBD, EUS-BD, and EUS-RV following ERCP failure and the effectiveness of CS-EHL for difficult CBDS extraction in our hospital. The secondary aim was to examine the safety of these procedures.

**Materials and methods:**

We conducted a retrospective evaluation of all the EUS-GBD, PT-GBD, EUS-BD, EUS-RV, and CS-EHL procedures, which were prospectively collected in the gastroenterology and digestive endoscopy unit and the general surgery unit from January 2020 to June 2023. The efficacy was expressed in terms of technical and clinical success rates, while safety was assessed based on the rate of adverse events (AEs).

**Results:**

We enrolled 83 patients with AC and high surgical risk. Among them, 57 patients (68.7%, 24/57 male, median age 85 ± 11 years) underwent EUS-GBD, and 26 (31.3%, 19/26 male, median age 83 ± 7 years) underwent PT-GBD. The technical and clinical success rates were 96.5 and 100% for EUS-GBD, and 96.1 and 92% for PT-GBD. The AEs for EUS-GBD were 1.7%, and for PT-GBD, it was 12%. ERCP for CBDS extraction failed in 77 patients. Among them, 73 patients (94.8%) underwent EUS-RV with technical and clinical success rates of 72.6% (53/73) and 100%, respectively. No AEs were reported. Four out of 77 patients were directly treated with EUS-BD for pyloric inflammatory stenosis. In 12 patients (16.4%), following unsuccessful EUS-RV with a CBD diameter ≥ 12 mm, an EUS-BD was performed. Both technical and clinical success rates for EUS-BD were 100%, and no AEs were reported. EUS-GBD was the treatment of choice for the remaining 8 (10.9%) patients after failure of both ERCP and EUS-RV. The procedure had high technical and clinical success rates (both at 100%), and no AEs were reported. The 12 difficult CBDS extraction treated with CS-EHL also showed high technical and clinical success rates (both at 100%), with no reported AEs.

**Conclusion:**

The minimally invasive approach for cholecystocholedocholithiasis, especially EUS-guided procedures, had high efficacy and safety in treating AC in high-risk surgical patients and CBDS extraction after a previously unsuccessful ERCP.

## 1 Introduction

Cholecystocholedocholithiasis refers to the presence of stones in both the gallbladder (GB) and the common bile duct (CBD).

Gallstones are common, particularly in Western countries (1–3), and it was estimated that 1–15% of patients with cholelithiasis also had CBD stones (CBDSs) (4–6).

The majority of patients with GB stones remain asymptomatic throughout their lifetime (7), and their annual risk of developing symptomatic disease (acute calculous cholecystitis (AC)) is approximately 2–3% (8). The occurrence of symptomatic disease and complications is mostly related to the migration of stones into the CBD.

According to guidelines, the gold standard treatment for CBDSs, whether symptomatic or not, is the extraction of stones through endoscopic retrograde cholangiopancreatography (ERCP) (9), and the gold standard treatment for AC is cholecystectomy (10, 11).

However, the treatment of cholecystocholedocholithiasis should be chosen based on the patient’s characteristics and the degree of severity of the disease.

In the case of patients not suitable for surgery, defined as “high surgical risk” (12, 13) [based on their comorbidities evaluated with the Charlson comorbidity index (12) and their health status before surgery estimated by the American Society of Anaesthesiologists’ Physical Status (ASA-PS) classification (13)], and not responsive to medical treatment, the guidelines recommend urgent or early GB drainage (10, 11).

Various types of GB drainage include percutaneous gallbladder drainage (PT-GBD), endoscopic transpapillary gallbladder drainage (ET-GBD), and endoscopic ultrasound (EUS)-guided gallbladder drainage (EUS-GBD).

While the majority of ERCP procedures are successful, selective biliary cannulation fails in 5–15% of cases, even in expert high-volume centers (14).

Despite advancements and new developments in endoscopic accessories, such as catheters, guidewires, stents, and sphincterotomes, ERCP failure can result from patients’ altered anatomy or difficult CBDS extraction.

In the event of ERCP failure, we have a variety of alternatives. Specifically, the EUS-guided approach is established as a viable alternative to ERCP.

EUS-guided rendezvous (EUS-RV) or EUS-guided biliary drainage (EUS-BD) is widely performed when conventional ERCP is not successful or not feasible due to various constraints (15).

Other alternatives for cases of ERCP failure are percutaneous transhepatic cholangiography and laparoscopic-endoscopic rendezvous, which combine a minimally invasive endoscopic approach with surgery in a single-stage operation.

Furthermore, the recent introduction of peroral cholangioscopy (CS)-guided electohydraulic lithotripsy (CS-EHL) has facilitated the management of difficult CBDS extraction.

So the minimally invasive approach for cholecystocholedocholithiasis is increasingly gaining recognition and validation in daily clinical practice. It not only supports surgery but also serves as a viable alternative to surgery itself in selected cases (13–16).

The primary aim of our study was to evaluate the efficacy, in terms of technical and clinical success rates, of EUS-GBD and PT-GBD in patients with AC who are unfit for surgery. We also aimed to evaluate the effectiveness of EUS-GBD, EUS-BD, and EUS-RV after ERCP failure, along with the efficacy of CS-EHL for difficult CBDS extraction in our gastroenterology and general surgery units.

The secondary aim was to evaluate the safety of all these minimally invasive treatments for cholecystocholedocholithiasis.

## 2 Materials and methods

We retrospectively evaluated all the EUS-GBD, PT-GBD, EUS-BD, EUS-RV, and CS-EHL procedures prospectively collected in the gastroenterology and digestive endoscopy unit and general surgery unit from January 2020 to June 2023.

All the included patients were ≥ 18 years old. Patients who underwent EUS-GBD or PT-GBD had a diagnosis of AC according to Tokyo Guidelines (17) and were classified as “high surgical risk” based on their comorbidities, evaluated with the Charlson comorbidity index (12), and their health status before surgery, estimated by the American Society of Anaesthesiologists’ Physical Status (ASA-PS) classification (13).

We performed EUS-GBD, EUS-BD, and EUS-RV for CBDS treatment following ERCP failure (18). The presence of CBDSs was confirmed before the endoscopic procedure using abdominal computed tomography, magnetic resonance cholangiopancreatography, or EUS.

ERCP failure was defined as unsuccessful CBD cannulation (the inability to gain deep and unobstructed access to the CBD) despite employing the double guidewire technique, precut papillotomy, fistulotomy, or transpancreatic sphincterotomy.

After experiencing at least two ERCP failures for benign biliary obstruction, we usually attempted EUS-RV first. If EUS-RV was unsuccessful and the CBD diameter was ≥12 mm, we performed EUS-BD, and if the CBD was <12 mm, and the patients had not undergone a previous cholecystectomy, EUS-GBD was done. In the event of EUS-RV failure in patients with a CBD < 12 mm and a history of cholecystectomy, there was an indication for percutaneous transhepatic cholangiography.

EHL during CS was performed for difficult CBDSs. “Difficult” biliary stones were defined based on their diameter (>1.5 cm), number, unusual shape (barrel shaped), or location (intrahepatic, cystic duct), or due to anatomical factors (narrowing of the bile duct distal to the stone, sigmoid-shaped CBD, stone impaction, shorter length of the distal CBD, or acute distal CBD angulation <135°) (9).

Patients who had previously failed biliary stone clearance were enrolled after a standard ERCP procedure with attempted stone removal using conventional techniques, such as stone extraction baskets or balloons, mechanical lithotripsy baskets, or endoscopic papillary large balloon dilation.

Before the procedure, patients received antibiotic prophylaxis based on local standards of care or the discretion of the endoscopist.

Patients under 18 years old with malignant biliary obstruction were not included in our study.

The efficacy of the minimally invasive approach for AC in patients unfit for surgery, those who had ERCP failure, and those who experienced difficult CBDS extraction was evaluated in terms of technical and clinical success rates.

Technical success was defined as the successful deployment of the lumen-apposing metal stent (LAMS) or the pigtail into the GB in patients with AC who are unfit for surgery. In cases of failed ERCP or complex CBDSs, the technical success was defined as CBDS extraction.

Clinical success was defined as the resolution of clinical symptoms of AC (e.g., fever, abdominal pain, and leukocytosis) and the resolution of cholestasis within 3 days after the procedure.

Safety was characterized by the occurrence of adverse events (AEs) (e.g., bleeding, malemployment, and death). The monitoring of the occurrence of AEs continued throughout the procedure and for the 72 h afterward. We defined “early AEs” as those occurring within the first 24 h after the procedure, while we defined “late AEs” as those that encompassed all AEs that occurred after 24 h after the procedure.

Baseline characteristics of patients, types of procedure, and procedural outcomes were summarized using means (SD) or medians (with interquartile range [IQR] and range) for continuous data, and frequencies and proportions for categorical data.

Datasets were compiled using Microsoft Excel, and all statistical analyses were performed using SPSS version 21 (IBM Corp., Armonk, New York, USA).

### 2.1 EUS-GBD

**Figure 1:**
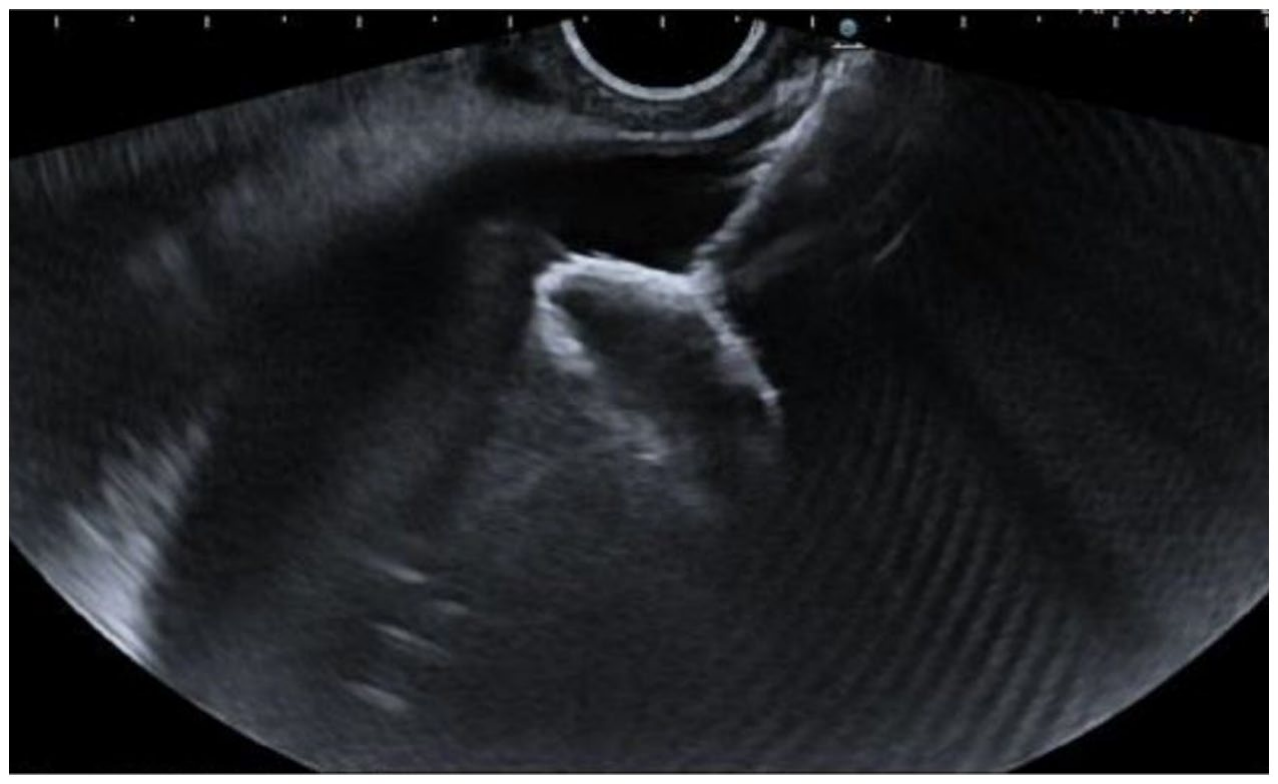
Deployment of the distal flange of the LAMS in the GB under EUS guidance.

**Figure 2:**
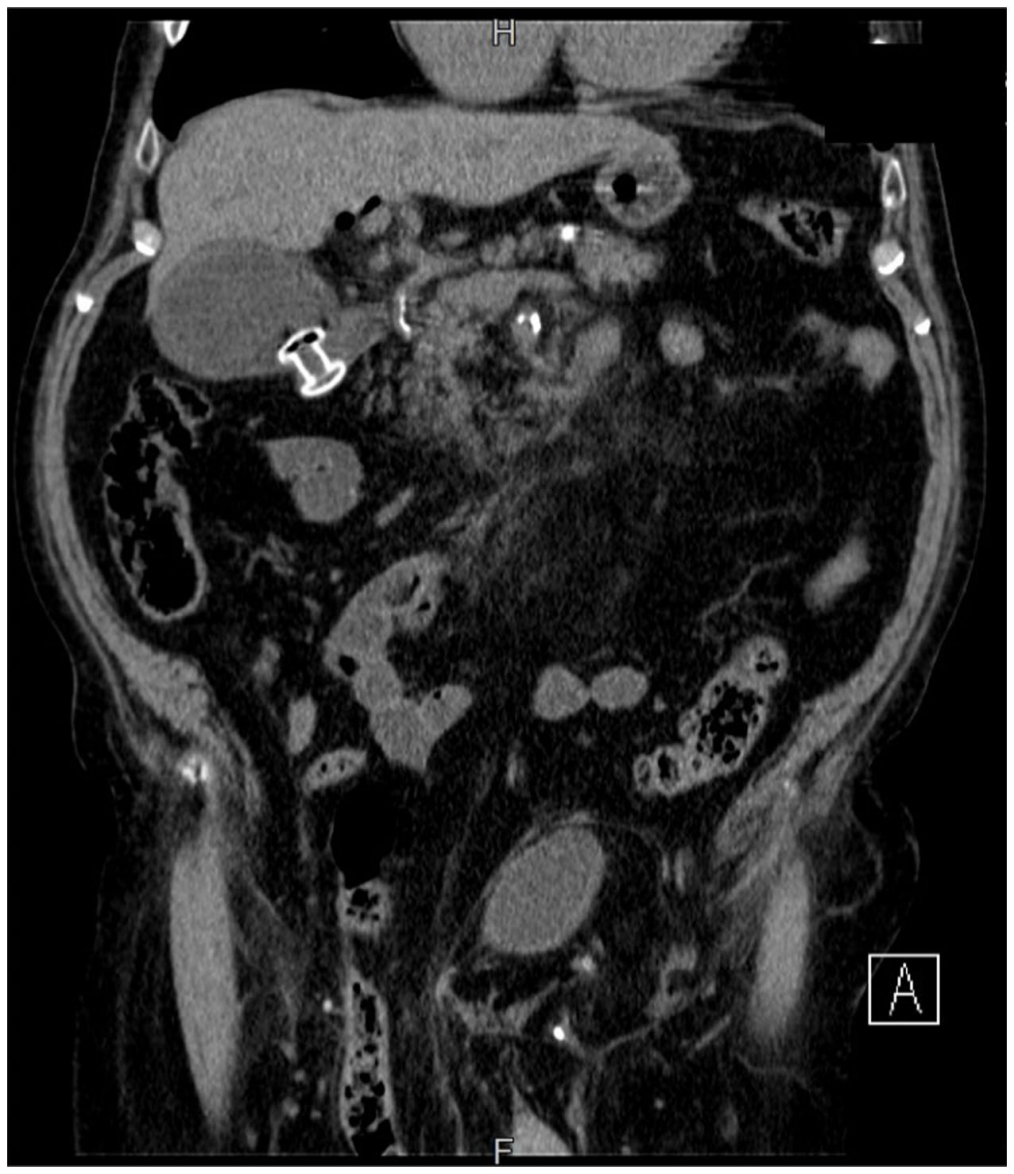
Abdominal computed tomography of EUS-GBD.

We used a slim, linear ultrasound endoscope (EG38-J10EGU, Pentax Medical, Germany) and the Arietta 70 ultrasound system. The transmural EUS-GBD approach involves placing a LAMS from the duodenum or stomach into the GB under EUS guidance (Figure 1), allowing, by the adherence of the GB to the gastrointestinal wall, the creation of a permanent fistulous tract (Figure 2).

The types of LAMS used were the Hot AXIOS stent (Boston Scientific, USA) or the Hot SPAXUS stent (Taewoong Medical, South Korea). The AXIOS stent sizes used were 15 mm × 10 mm and 10 mm × 10 mm, while the SPAXUS stent size was 16 mm × 20 mm.

### 2.2 PT-GBD

After visualizing the GB under ultrasound (US) guidance and administering the anesthetic infiltration into the peritoneum at the puncture site, the GB was punctured using a disposable pigtail drainage catheter under US guidance. The puncture needle was directed into the gallbladder cavity, and the outflow of bile was observed. Then, a guidewire was inserted into the GB, and the drainage tube was placed into the GB lumen over the guidewire. A drainage bag was then fixed at the drainage tube. Finally, after fixing the drainage tube, the body surface was sutured. Following the procedure, routine anti-infection, semiliquid, low-fat food, and other symptomatic and supportive treatments were given.

### 2.3 EUS-RV

We used a duodenoscope (TJF-Q190V, Olympus), a slim, linear ultrasound endoscope (EG38-J10EGU, Pentax Medical, Germany), and the Arietta 70 ultrasound system.

Under EUS guidance, the CBD was punctured with a 19-gauge EUS-guided FNA needle from either the stomach or the duodenal bulb. Following contrast injection in the CBD to confirm the correct position, a guidewire was passed into the CBD through the EUS needle and then manipulated through the papilla into the descending duodenum. The guidewire was then left in place, and a duodenoscope was maneuvered to the second portion of the duodenum; the wire was used to facilitate ampullary cannulation, and a conventional ERCP could then be performed.

### 2.4 EUS-BD

**Figure 3:**
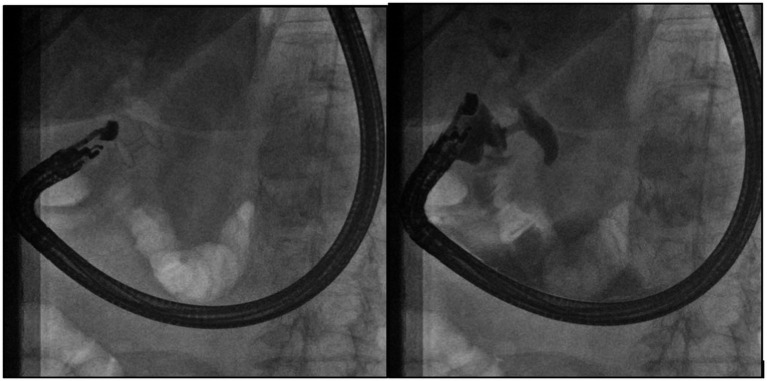
Abdominal computed tomography of EUS-GBD. In the left image, the LAMS was deployed between the duodenum and CBD. In the right image, the contrast flowed thorough the LAMS into the CBD.

We used a slim, linear ultrasound endoscope (EG38-J10EGU, Pentax Medical, Germany) and the Arietta 70 ultrasound system. Under EUS guidance, a LAMS was deployed between the CBD lumen and the gastric or duodenal lumen. At the end of the procedure, the correct LAMS deployment was confirmed by contrast injection through the LAMS lumen into CBD (Figure 3).

We utilized AXIOS stent sizes of 8 mm × 8 mm and 6 mm × 8 mm.

### 2.5 CS-EHL

CS was performed using the SpyGlass Direct Visualization System (Boston Scientific, Marlborough, Massachusetts, USA), and EHL was conducted using the Autolith Touch EHL System (Boston Scientific, Marlborough, Massachusetts, USA). CS was performed following sphincterotomy or balloon sphincteroplasty.

EHL systems worked through a bipolar probe and a charge generator; transmitting a charge across the electrodes at the tip of the probe generated a spark. This determined the expansion of the surrounding fluid and finally resulted in an oscillating shock wave of pressure that fragmented the stones. Under direct visualization, the EHL probe was directed at the stone positioned at least 5 mm from the tip of the cholangioscope and 1–2 mm from the stone (19)

Thus, shock wave technology under CS guidance allowed the fragmentation of large and challenging biliary stones.

## 3 Results

We retrospectively collected data from a total of 172 patients over a span of 42 months from the gastroenterology and digestive endoscopy unit and the general surgery unit of our hospital.

Among all the patients, 83 (48.3%) had a diagnosis of AC and were considered unfit for surgery due to their high surgical risk resulting from comorbidities; 77 (44.8%) patients had a previous failed ERCP, and 12 (6.9%) had difficult CBDS extraction.

### 3.1 AC in patients unfit for surgery

A total of 83 patients had a diagnosis of AC, and they could not undergo cholecystectomy due to their high surgical risk. Among them, 43 (51.8%) patients were male with a median age of 84 ± 9 years.

EUS-GBD was performed in 57 out of 83 (68.7%) patients unfit for surgery, while the remaining 26 out of 83 (31.3%) underwent PT-GBD.

Among the 57 out of 83 (68.7%) patients with AC deemed unfit for surgery and treated with EUS-GB, 24 (42.1%) patients were male with a median age of 85 ± 11 years.

In 47 (82.5%) EUS-GBD procedures, a 10 mm × 10 mm LAMS was deployed; in 8 (14%) cases, a 15 × 10 mm LAMS was used, and in two (3.5%) patients, a 16 × 20 mm LAMS was utilized.

The transduodenal approach was performed in 43 out of 57 (75.4%) cases, while in 14 out of 57 (24.6%), LAMS was deployed through the stomach.

Technical success was achieved in 55 (96.5%) patients, and clinical success was attained in all 55 (100%) cases with the correct deployment of the LAMS in the GB. In two (3.5%) patients, the EUS-GBD was not correctly performed: in one patient, the distal flange of the LAMS was accidentally deployed in the bladder lumen, and in the other patients, the EUS-GBD was not feasible due to the inability to identify a correct and secure position for LAMS deployment.

The patients with the LAMS positioned between the stomach and the bladder underwent emergency surgery. The LAMS was removed, and the walls of the stomach and bladder were sutured.

Only in one (1.7%) case of EUS-GBD, an AE was registered: there was intraprocedural bleeding, which was self-limited after LAMS deployment.

A total of 26 out of 83 patients underwent PT-GBD [19 (73%) being male, and the median age was 83 ± 7 years]. The double pigtail used had a size of 9 Fr.

The technical success was achieved in 96.1% (*n* = 25/26), and the clinical success was observed in 92% (*n* = 23/25).

In two patients (8%), there was no improvement in their clinical conditions. So one patient was referred for surgery to undergo cholecystectomy, and the other patient underwent EUS-GBD, resulting in subsequent clinical improvement in both cases.

The early AE rate was 4% (1/25): in one patient, peritonitis was registered after PT-GBD, leading to subsequent GB rupture. Late AEs were reported in two cases (8%) since there was double pigtail dislodgement. The total PT-GBD AE rate was 12%.

The characteristics of patients who underwent EUS-GBD or PT-GBD are summarized in Table 1.

**Table 1:** Characteristics of patients unfit for surgery undergone to EUS-GBD and PT-GBD for acute cholecystitis.

	*N* (%)
EUS-GBD	57/83 (68.7%)
Gender (male)	24 (42.1%)
Age (median)	85 ± 11 years
LAMS 16 × 20 LAMS 15 × 10 LAMS 10 × 10	2 (3.5%) 8 (14%) 47 (82.5%)
Trans-gastric Transduodenal	14 (24.6%) 43 (75.4%)
Technical success Clinical success	55 (96.5%) 55 (100%)
Adverse events	1 (1.7%)
PT-GBD	26/83 (21.3%)
Gender (male)	19 (73%)
Age (median)	83 ± 7 years
Technical success Clinical success	25 (96.1%) 23 (92%)
Adverse events	3 (12%)

EUS-GBD: endoscopic ultrasound-guided gallbladder drainage.

PT-GBD: percutaneous gallbladder drainage.

### 3.2 Failed ERCP

We collected data from a total of 77 patients with CBDSs and ERCP failure. Among them, 73 (94.8%) underwent an EUS-RV after ERCP failure, and in only four (5.2%) cases, immediate EUS-BD was performed due to inflammatory pyloric stenosis, which made the descending duodenum inaccessible.

The 73 patients who underwent EUS-RV after ERCP failure had a median age of 81 ± 11 years, and 56.2% (*N* = 41/73) were male. CBDS extraction was achieved in 53 out of 73 (72.6%) patients with EUS-RV. In these patients, the clinical success rate was 100%, and no AE was reported.

The main cause of EUS-RV failure was the fact that the guidewire did not pass through the papilla of Vater or that the guidewire was not correctly oriented toward distal CBD.

Following unsuccessful EUS-RV, in 12 (16.4%) patients with a CBD diameter ≥ 12 mm, an EUS-BD was performed. Thus, a total of 16 patients (12/16 after EUS-RV failure and 4/16 for inflammatory pyloric stenosis) underwent EUS-BD. Among them, nine (56.2%) patients were male, and the median age was 78.5 ± 13.7 years. Both the technical and clinical success rates were 100%, and no AE was reported.

In 8 out of 73 (10.9%) patients, an EUS-GBD was performed after EUS-RV failure. These patients had a CBD size <12 mm, with a median age of 84 ± 28 years, and 6 out of 8 (75%) were male.

Both the technical and clinical success rates were 100%, and no AE was reported.

All the characteristics of the patients with ERCP failure are documented in Table 2.

**Table 2:** Characteristics of patients undergone EUS-RV, EUS-BD, and EUS-GBD after ERCP failure.

	*N* (%)
EUS-RV	73/77 (94.8%)
Gender (male)	41 (56.2%)
Age (median)	81 ± 11 years
Technical success Clinical success	53 (72.6%) 53 (100%)
Adverse events	0 (0%)
EUS-BD after ERCP failure EUS-BD after EUS-RV failure	4/77 (5.2%) 12/20 (60%)
Gender (male)	9 (56.2%)
Age (median)	78.5 ± 13.7 years
Technical success Clinical success	16 (100%) 16 (100%)
Adverse events	0 (0%)
EUS-GBD after EUS-RV failure	8/20 (40%)
Gender (male)	6 (75%)
Age (median)	84 ± 28 years old
Technical success Clinical success	8 (100%) 8 (100%)
Adverse events	0 (0%)

EUS-RV: endoscopic ultrasound-guided rendezvous.

EUS-BD: endoscopic ultrasound-guided biliary drainage.

EUS-GBD: endoscopic ultrasound-guided gallbladder drainage.

### 3.3 Difficult CBDS extraction

A total of 12 cases with difficult CBDS extraction were treated with CS-EHL. In 11 (91.6%) cases, a single session of CS-EHL was sufficient to achieve the complete CBDS extraction; only in one case, a double session was necessary to obtain complete stone extraction.

The mean age of the patients was 77 ± 8 years, and in 8 out of 12 (66.7%) cases, the patients were male.

Both the technical and clinical success rates were 100%, and no AE was reported.

## 4 Discussion

The minimally invasive treatment of cholecystocholedocholithiasis had become increasingly integrated into daily clinical practice to the extent that the major guidelines (10, 11) recommended GB drainage for patients with AC who were unfit for surgery and non-responsive to medical therapy.

While Tokyo Guidelines (10) suggested PT-GBD, WSES guidelines (11) recommended endoscopic transpapillary gallbladder drainage (ETGBD) and EUS-GBD as safe and effective alternatives to PT-GBD.

Furthermore, WSES guidelines (11) suggested that EUS-GBD with LAMSs should be preferred to ETGBD when performed by a skilled endoscopist.

Although PT-GBD had been the most common non-surgical treatment for GB decompression for years, it showed significant morbidity (50–75%) (20) and a recurrent cholecystitis rate of up to 15.4% (21). Moreover, when compared to EUS-GBD, PT-GBD exhibited comparable technical and clinical success rates (22–25) but was associated with a longer hospital stay, longer time for clinical resolution, and higher rates of reintervention and recurrent AC (18–20).

Multicentre studies also showed that PT-GBD was associated with higher post-procedural pain and more AEs at both 30 days (18, 26) and 1 year (19) compared to EUS-GBD.

In the literature, EUS-GBD was reported to have very high technical and clinical success rates, ranging from 90 to 98.7% and 89 to 98.4%, respectively (18, 20, 27–31).

Our data, in terms of EUS-GBD efficacy, were similar to those reported in the literature, with technical and success rates of 96.5 and 100%, respectively, in our units.

Considering AE rate, EUS-GBD, when compared to PT-GBD and ETGBD, showed a significantly lower AE rate (14.6% versus 30% for PT-GBD) (24) and had the lowest risk of recurrent AC (32).

In our single-center case series, the AE rate for EUS-GBD was 1.7%, while that for PT-GBD was 12% (4% early AEs and 8% late AEs).

Although ERCP had been the gold standard treatment for CBDSs and numerous improvements were made to ERCP devices over the years, unsuccessful biliary cannulation could always happen. According to the literature, biliary cannulation failed in 5–15% of cases (12) and, in our unit, we reported an ERCP failure rate of 4.7%. In such cases, performing EUS-RV could be a viable option to allow biliary cannulation.

A literature review showed that EUS-RV had an overall success rate of 82% (33) and the main reason for EUS-RV failure was the inability to guide the wire in the direction of the CBD.

In our unit, the technical success of EUS-RV was 72.6%, which is lower than the data reported in the literature, as our protocol included the execution of a few attempts to overcome the papilla with the guidewire. Subsequently, the guidewire was quickly employed to guide the deployment of the LAMS for EUS-BD.

Over time, with development and technical improvement, EUS-BD became more effective and safer. A systematic review published in 2016 by Wang et al. (34) showed higher technical success rates of EUS-BD in studies conducted from 2013 onward.

While, in cases of malignant distal CBD obstruction, EUS-BD showed a technical success rate of up to 95% (35, 36) its performance for benign pathology has been less extensively studied.

EUS-BD was usually performed after failed ERCP in patients with surgically altered anatomy.

Although, in such cases, the traditional salvage therapy was percutaneous biliary drainage, a recent survey demonstrated that many patients would prefer internal biliary drainage to an external drain (37).

The initial studies published about EUS-BD (both choledochoduodenostomy and hepaticogastrostomy) for CBDS treatment showed a cumulative success rate ranging from 60 to 72% (38–40) whereas three recent studies demonstrated notable improvement in procedural and clinical success rates ranging from 91.9 to 100% (41–43).

In our unit, the technical and clinical success rates for EUS-BD in benign pathology were high (both at 100%), and they overlapped, if not surpassed, the outcomes showed in these three recent studies.

Moreover, no AEs were reported in our case series.

When CBDS extraction or biliary stricture access was difficult due to limited biliary ductal dilation or long distances for the guidewire to traverse, a two-step EUS-guided drainage approach was recently proposed.

In this method, the first step involved stent placement (typically with EUS-guided hepaticogastrostomy), followed by antegrade stone extraction once the fistula matures (44). One of the initial pilot studies using this approach in seven patients with anastomotic strictures reported clinical and technical success rates of 100 and 57%, respectively (45).

However, at this point in time, although EUS-BD was becoming a reliable alternative to ERCP and percutaneous biliary drainage (46), there were currently no head-to-head studies comparing ERCP to EUS-BD for the treatment of benign biliary pathology. As more studies are conducted, the clinical success and safety profile of the two-step approach will likely catapult EUS-guided intervention as a potential first-line tool in cases of surgically altered anatomy (47). However, standardization of the procedure with dedicated devices was still needed.

Possibly, EUS-BD may soon be established as an effective therapeutic option in the initial approach or indicated in cases where there were predictors of difficult ERCP (i.e., difficult biliary cannulation, tumor invasion of the papilla, and duodenal obstruction) (48).

In cases of large CBDSs that were challenging to grasp due to breakup using a mechanical lithotripter or in those with bile duct strictures, CS-EHL was particularly effective. We reported a technical and clinical success rate of 100% without registering any AEs. These data were similar to those showed in the literature (49), although a higher AE rate was reported (up to 24.2% in patients ≥80 years and up to 17.5% in non-elderly) (50).

The limitations of our study were attributed to its retrospective nature and the relatively small number of cases.

In conclusion, ERCP retained its status as the treatment of choice for CBDS extraction. However, the EUS-guided approach (EUS-RV, EUS-BD, and EUS-GBD) was a possible and effective alternative in cases of failed ERCP. They became attractive due to their technical simplicity, preservation of hepatic parenchyma, potential performing ability in ascitic patients, and lower complication rate (51, 52). However, further studies were needed to evaluate the EUS-guided approach for the treatment of CBDSs.

However, the EUS-guided approach for the treatment of AC was demonstrated to be a safe and effective procedure in patients at high surgical risk.

## 5 Conclusion

The minimally invasive approach for cholecystocholedocholithiasis, especially EUS-guided procedures, had high efficacy and safety in treating AC in high-risk surgical patients and CBDS extraction following a previous failed ERCP.

## Data availability statement

The raw data supporting the conclusions of this article will be made available by the authors, without undue reservation.

## Ethics statement

Ethical approval was not required for the studies involving humans because it is a retrospective analysis, and informed consent is not required. The studies were conducted in accordance with the local legislation and institutional requirements. Written informed consent for participation was not required from the participants or the participants’ legal guardians/next of kin in accordance with the national legislation and institutional requirements because it is a retrospective analysis.

## Author contributions

AC: Conceptualization, Methodology, Project administration, Writing – original draft. GioA: Writing – review & editing. GC: Writing – review & editing. GiaA: Writing – review & editing. PC: Writing – review & editing. FB: Conceptualization, Methodology, Validation, Writing – review & editing.
